# Coronary Heart Disease in Postmenopausal Women with Type II Diabetes Mellitus and the Impact of Estrogen Replacement Therapy: A Narrative Review

**DOI:** 10.1155/2014/413920

**Published:** 2014-07-17

**Authors:** Marouane Boukhris, Salvatore Davide Tomasello, Francesco Marzà, Sonia Bregante, Francesca Romana Pluchinotta, Alfredo Ruggero Galassi

**Affiliations:** ^1^Department of Medical Sciences and Pediatrics, Catheterization Laboratory and Cardiovascular Interventional Unit, Cannizzaro Hospital, University of Catania, Via Messina 829, 95126 Catania, Italy; ^2^IRCCS Policlinico S. Donato, Via Morandi 30, 20097 Milano, Italy

## Abstract

Coronary heart disease is the main cause of death in postmenopausal women (PMW); moreover its mortality exceeds those for breast cancer in women at all ages. Type II diabetes mellitus is a major cardiovascular risk factor and there is some evidence that the risk conferred by diabetes is greater in women than in men. It was established that the deficiency of endogenous estrogens promotes the atherosclerosis process. However, the impact of estrogen replacement therapy (ERT) on cardiovascular prevention remains controversial. Some authors strongly recommend it, whereas others revealed a concerning trend toward harm. This review tries to underlines the different components of cardiovascular risk in diabetic PMW and to define the place of ERT.

## 1. Introduction 

The mortality attributable to coronary artery disease (CAD) exceeds breast cancer mortality in women at all ages [1]; however CAD is the main cause of death in postmenopausal women (PMW) [2]. It is beyond doubt that the cardiovascular risk is multifactorial and several factors come into play, but such a difference has been attributed to the protective effects of female sex hormones, particularly estrogens, before menopause [3]. Type II diabetes mellitus (DM) is a major risk factor for myocardial infarction (MI) and CAD [4–6]. There is some evidence that the risk conferred by DM is greater in women than in men [7]; indeed, in a 13-year prospective study, the incidence of major cardiovascular events in subjects without DM was roughly sixfold greater in men than in women. In presence of DM, the gender difference was lost [8]. Literature about the impact of estrogen replacement therapy (ERT) on the cardiovascular risk is controversial. Some authors supported a protective cardiovascular benefit of ERT after menopause [9], while randomized placebo-controlled trials carried out in both primary [10] and secondary [11] preventions showed a concerning trend toward harm. For all these reasons many questions remain unanswered. This review tries to underline the different components of cardiovascular risk in diabetic PMW and to define the place of ERT.

## 2. Cardiovascular Risk in Postmenopausal Women 

With age, women become more likely to develop type 2 DM: at the age of 50–59 years, approximately 12.5% of women have a known type 2 DM; at the age of 60 years and older, the rate increases to 17-18% (a 25–30% increase). Moreover, type 2 DM remains undiagnosed in more than one-third of these women [12]. Diabetic PMW are three times more likely to develop CAD or stroke than nondiabetic women [13–15]. Furthermore, a diabetic woman is four times more likely to die from MI than a diabetic man [16].

The increased rate of CAD in PMW seems related, in part, to the loss of the protection offered by endogenous estrogen. This finding is supported by the dramatic increase in CAD seen in women after surgically induced menopause [17]. On the other hand, a greater incidence of hypertension and hyperlipidemia as well as an elevated body mass index is observed after menopause [18].

With age, the body of a PMW tends to lose lean body tissue and gain in adipose tissue, particularly in abdominal location [19]. The sedentary lifestyle that often accompanies aging may also contribute to obesity. As a consequence, the insulin resistance increases with its linked dyslipidemia and coagulation abnormalities [19]. In fact, the insulin resistance state due to DM is responsible for an increased hepatic synthesis of triglyceride- (TG-) rich lipoproteins and a faster clearance of high-density lipoproteins cholesterol (HDL-C) [20–22]. Therefore, the dyslipidemia in postmenopausal diabetic women is characterized by elevated plasma TG, reduced HDL-C, and elevated small low-density lipoprotein (LDL) serum levels [23]. Abnormalities in coagulation and fibrinolysis are often seen in type 2 DM including cardiovascular risk indicators such as fibrinogen, factor VII, von Willebrand factor, tissue type-plasminogen activator antigen, and plasminogen activator inhibitor-1 (PAI-1) antigen and activity [24–27]. In addition, peri- and postmenopausal increase in coagulation [28] and decrease in fibrinolysis [29] have been described. Although it has been shown that premenopausal women produce significantly less thromboxane B2 than age-matched men, women show a linear increase in the level of the prostaglandin during the postmenopausal years, whereas such an increase has not been found in men [30].

Menopause is associated with an increase in blood pressure (BP) and a decrease in physiologic nocturnal BP fall [31]. Furthermore, diabetic subjects have increased vascular load and abnormal 24 h BP profiles [32]. These factors may play a role in the increased risk of cardiovascular events in diabetic PMW.

Approximately 25% of PMW smoke cigarettes [33]. Cigarette smoking is associated highly with cardiovascular disease, and in women, it is estimated that 21% of all mortality from cardiovascular disease is related to cigarette smoking [33]. Oncken et al. [34] found that smoking cessation in PMW decreases systolic BP by 3.6 ± 1.9 mm Hg and awake heart rate by 7 ± 1 beats/min. These hemodynamic changes are due in part to reductions in sympathetic nervous system activity [34].

DM is also associated with a diminished nitric oxide bioavailability. This promotes atherogenesis through decreased leukocyte adhesion, increased platelet aggregation, and increased vascular smooth muscle growth [35]. This also can cause constriction of coronary arteries during physical or emotional stress, contributing to myocardial ischemia [36]. Women may have false-positive treadmill electrocardiographs with normal coronary angiograms. The so-called syndrome X combining typical angina, ST segment depression, and normal coronary angiography is much more common in women with estrogen deficiency [37, 38]. Compared with men, women had more symptoms and less anatomic coronary artery disease at baseline, with persistence of higher angina rates with or without prompt revascularization [39].

In a retrospective analysis of the Women's Angiographic Vitamin and Estrogen (WAVE) trial, a multicenter randomized trial on progression of atherosclerosis in PMW, Ahmad et al. [40] found a complex relation between DM and the progression of CAD in PMW. In fact, clinically apparent DM, not elevated glycosylated hemoglobin (HbA1c) alone, appears to promote the progression of established coronary lesions even in low HbA1c rates. This raises the possibility that coronary narrowing of existing stenosis in diabetic women may be due to negative remodeling, a complex process that might be less dependent on hyperglycemia than new lesion formation. There has been considerable interest regarding the importance of sex in contributing to mortality rate after percutaneous coronary revascularization. The 1985-1986 National Heart, Lung, and Blood Institute's Coronary Angioplasty Registry documented a hospital mortality rate of 2.6% in women versus 0.3% in men. Some of the difference was related to the fact that women were older and had a higher risk: congestive heart failure, diabetes, and multivessel disease. However, even after adjusting for these parameters, women had a significantly higher mortality rate [41]. Bell et al. reported the same results in the Mayo Clinic experience with 3557 coronary interventions from 1979 to 1990 [42]. Despite the improvement in the management of MI and complications [43, 44], the long-term prognosis remains unsatisfactory in this subsets of patients, particularly diabetics [45, 46]. In fact, DM is associated with impaired perfusion and distal embolization, which contribute to explaining the higher mortality [47]. Therefore, it is difficult to predict the prognosis of percutaneous revascularization in diabetic PMW. Complete revascularization, when possible, is recommended [48]. Second generation of drug-eluting stents is generally preferred [49] and it was shown that diabetic women required larger stents than diabetic men [50]. It is notable that coronary artery bypass surgery in women is more cumbersome in comparison with males, requiring longer intubation times, intensive care unit length of stay, and hospital length of stay [51]. Arterial grafts are recommended. Indeed, the use of a radial artery graft has been proven to improve survival compared with use of a saphenous vein graft [52].

## 3. Cardiovascular Effects of Estrogen Replacement Therapy 

Estrogen has favorable impact on the risk factors of atherosclerosis and therefore CAD [53]. The inflammatory process in atherosclerosis involves a large group of factors and molecules [54]. Growth factors and cytokines play a central role in the development of atherosclerosis [55]. The presence of estrogen receptors, found on human monocytes, suggests that estrogen may modulate the release of such molecules [56]. Patients who had undergone a complete hysterectomy showed higher levels of interleukin 1 (IL-1) activity than postmenopausal women who were under hormone replacement therapy [57]. Aune et al. found that after 12 months of a hormone replacement therapy (HRT), the levels of tumor necrosis factor (TNF*α*) produced by lipopolysaccharide-stimulated macrophages had decreased significantly in both patients receiving estrogen orally and those receiving treatment transdermally [58]. By studying the levels of cytokines in postmenopausal women on HRT and those who were not on a therapy, Kamada et al. [59] found that the women on HRT had a significant increase in colony-stimulating factor, which is known to decrease serum cholesterol.

Although there is some concern over its tendency to increase TG serum levels [60], it was also well established that estrogen administration reduces levels of LDL and increases HDL in PMW, thus restoring the lipid profile back to premenopause state [61]. In rabbit aortas, estrogen therapy was also able to decrease collagen production reducing the progression of atherosclerotic plaque [62]. Many studies have been focused on the relation between estrogen, blood coagulation, and the formation of emboli [63, 64]. Estrogen has two completely opposing effects: proinflammatory effect with D-dimer, metalloproteinase 9 and factor VII and anti-inflammatory effect found with fibrinogen, endothelial adhesion molecules, and plasminogen activator inhibitor-1 (PAI-1) [65, 66]. It is difficult to determine which effect will prevail.

Koh et al. studied the effects of HRT on the levels of PAI-1 (a potent inhibitor of fibrinolysis) and D-dimer (a by-product of fibrinolysis) [66]. D-dimer levels increased proportionately with decreasing PAI-1 levels. These findings showed the impact of estrogen on promoting fibrinolysis. In contrast, it has been demonstrated that estrogen also activates coagulation system [66]. By the administration of estrogen to healthy PMW, Caine et al. [67] noted a dose-dependent increase in thrombin generation and fibrinopeptide A. Furthermore, Scarabin et al. [68] found that an oral regimen of estrogen with cyclic progesterone increased levels of prothrombin fragments 1 and 2 and decreased antithrombin activity in healthy PMW. Lee et al. [69] have shown that the basal endothelium dependent vascular reactivity was significantly decreased in PMW with diabetes compared with normal PMW. Although estrogen supplementation increased endothelium dependent vasodilation not only in PMW with diabetes but also in normal PMW, the endothelial dysfunction was not entirely corrected. The etiology of vascular dysfunction in DM is still not fully understood. High levels of glucose may result in a dysregulation of endothelial nitric oxide synthase enzyme function responsible for a decrease in nitric oxide production [70].

Several hypotheses of the mechanism of vasodilation after estrogen treatment exist including the release of prostaglandin and nitric oxide and the activation of potassium or calcium channels [71–73]. However, such findings were not confirmed by Koh et al. [74] who found that the effects of estrogen on endothelial, vascular dilatory and other homeostatic functions were less apparent in type II diabetic postmenopausal women.

Otherwise, estrogen has also been shown to have some protective capabilities against ischemia-reperfusion injury in various organs. Squadrito et al. [75] exposed rats under 17 *β*-estradiol regimen and untreated rats to 1 h of left coronary artery occlusion followed by 1 h of reperfusion and found that the administration of estrogen 5 min after the induction of injury decreased the markers and the degree of necrosis. This may be due to the antioxidant effect of estrogen [76]. Indeed, Rifici and Khachadurian [77] described a capital role of 17 *β*-estradiol in the inhibition of LDL oxidation.

It has been reported that HRT decreases angiotensin converting enzyme activity, which may be one of the factors protecting against CAD. Sumino et al. [78] reported an increased level of bradykinin not only in hypertensive but also in nonhypertensive PMW. It has been also demonstrated that estrogen has calcium channel blocking effects [79, 80]. All these arguments are in favor of an antihypertensive role of estrogen. However, Hayward et al. [32] did not find any beneficial effect of HRT on indexes of arterial load and ambulatory BP in diabetic postmenopausal women.

## 4. Place of the Hormone Replacement Therapy in Cardiovascular Prevention in Postmenopausal Women 

It is difficult to predict the overall harm or benefit of HRT on the cardiovascular system considering the pleiotropic effects of estrogens due to the ubiquitous distribution of estrogen receptors in different organs and systems. In both Heart and Estrogen/Progestin Replacement Study (HERS) [11] and Women's Health Initiative (WHI) [10] trial, the increased risk of vascular and thromboembolic events was observed particularly when starting HRT. In WHI, this risk increased mostly within the 2 first years of HRT [10]. In HERS, the relative risk for coronary events was increased more than twofold within the first 4 months and also normalized within 2 years [11]. Otherwise, the effects of estrogen may vastly vary depending on the age, the other risk factors, and the stage of CAD [81]. As the risk of myocardial infarction increases sharply not before the late fifties followed by cerebrovascular disease a decade later, the results of neither HERS nor WHI can be in part transferred to the HRT prescription in peri- and early menopause. A report on more than 24,000 diabetic postmenopausal women revealed an increased rate of MI within the first year of HRT, only in women with a previous MI [82]. However to conclude that WHI only comprises healthy women is inadequate, since many women at the age of 60 will have silent ischaemia, especially those with DM.  Table 1 summarized the findings of some key studies focusing on the HRT impact on cardiovascular risk in PMW.

The administrated dose of estrogen has also a determining influence. In fact, in the Northern California Kaiser Permanente Diabetes, the most important decrease of cardiovascular risk was obtained with 0.3 mg conjugated estrogens or 2 mg ethinyl estradiol. With higher doses, this reduction vanished and even an elevated risk appeared [82].

In addition, it was clearly demonstrated that socioeconomic status, correlated with the use of HRT, influences the cardiovascular risk in postmenopausal women [83]. The characteristics of the women using HRT differ from those of the nonusers. Indeed, HRT users tend to have greater contact with the health care system. This issue is particularly capital in diabetics. For example, a diabetic PMW under HRT may have more chance to be better followed for her DM and earlier diagnosed for nephropathy, retinopathy, and CAD.

## 5. Conclusion 

The incidence and the mortality of CAD in women increase after menopause. The loss of ovarian function and the subsequent deficiency of endogenous estrogens, added to age, abdominal obesity and particularly DM, promote the atherosclerosis process. It is well established that estrogen has favorable effects on some of the major risk factors of CAD. However, only large clinical trials may help to decide which dose, for which women, and at which age HRT is beneficial or harmful. Finally, cardiovascular prevention requires correcting the lifestyle and other classical risk factors, particularly a strict control of diabetes, since estrogen alone cannot be expected to counteract the entire cardiovascular risk.

## Figures and Tables

**Table 1:** Summary and conclusions of some key studies focusing on the hormonal replacement therapy impact on cardiovascular risk in postmenopausal women.

Study	Year	Design of the study	*N* patients	HRT	Follow-up	Conclusions
Grodstein et al. [9]	1996	Prospective study	59,337	ERT alone or combined HRT	16 years	Current hormone users, regardless of whether they used ERT alone or combined HRT, tended to have a better risk profile than women who had never used HRT.

Hulley et al. [11] (HERS trial)	1998	Randomized, blinded, placebo-controlled secondary prevention	2,763	0.625 mg of conjugated equine oestrogens + 2.5 mg medroxyprogesterone acetate	4.1 years	HRT did not reduce the overall rate of CAD events in PMW with established CAD. Moreover, HRT did increase the rate of thromboembolic events and gallbladder disease.

Rossouw et al. [10] (WHI trial)	2002	Randomized placebo-controlled primary prevention trial	16,708	0.625 mg of conjugated equine oestrogens + 2.5 mg medroxyprogesterone acetate	5.2 years	Overall health risks exceeded benefits from use of HRT in healthy PMW. HRT should not be indicated for CAD primary prevention.

Ferrara et al. [82] (Northern California Kaiser Permanente Diabetes Registry)	2003	Survey follow-up study	15,435	ERT alone or combined HRT	3 years	In diabetic PMW without a recent MI, use of combined HRT was associated with decreased risk of MI. However, HRT was associated with increased risk of MI in women with history of a recent MI.

CAD: coronary artery disease; ERT: estrogen replacement therapy; HRT: hormonal replacement therapy; MI: myocardial infarction; PMW: postmenopausal women.
